# The Use of Ribosomal RNA as a Microbial Source Tracking Target Highlights the Assay Host-Specificity Requirement in Water Quality Assessments

**DOI:** 10.3389/fmicb.2021.673306

**Published:** 2021-06-02

**Authors:** Annastiina Rytkönen, Ananda Tiwari, Anna-Maria Hokajärvi, Sari Uusheimo, Asko Vepsäläinen, Tiina Tulonen, Tarja Pitkänen

**Affiliations:** ^1^Expert Microbiology Unit, Finnish Institute for Health and Welfare, Kuopio, Finland; ^2^Lammi Biological Station, Faculty of Biological and Environmental Sciences, University of Helsinki, Helsinki, Finland; ^3^Environmental Health Unit, Finnish Institute for Health and Welfare, Kuopio, Finland; ^4^Department of Food Hygiene and Environmental Health, Faculty of Veterinary Medicine, University of Helsinki, Helsinki, Finland

**Keywords:** microbial source tracking, performance analysis, field validation, ribosomal RNA, RT-qPCR, fecal contamination, surface water

## Abstract

For microbial source tracking (MST), the 16S ribosomal RNA genes (rDNA) of host-specific bacteria and mitochondrial DNA (mtDNA) of animal species, known to cause fecal contamination of water, have been commonly used as molecular targets. However, low levels of contamination might remain undetected by using these DNA-based qPCR assays. The high copy numbers of ribosomal RNA (rRNA) could offer a solution for such applications of MST. This study compared the performance of eight MST assays: GenBac3 (general *Bacteroidales*), HF183 (human), BacCan (dog), Rum-2-Bac (ruminant), Pig-2-Bac (swine), Gull4 (gull), GFD, and Av4143 (birds) between rRNA-based and rDNA-based approaches. Three mtDNA-based approaches were tested: DogND5, SheepCytB, and HorseCytB. A total of 151 animal fecal samples and eight municipal sewage samples from four regions of Finland were collected for the marker evaluation. The usability of these markers was tested by using a total of 95 surface water samples with an unknown pollution load. Overall, the performance (specificity, sensitivity, and accuracy) of mtDNA-based assays was excellent (95–100%), but these markers were very seldom detected from the tested surface water samples. The rRNA template increased the sensitivity of assays in comparison to the rDNA template. All rRNA-based assays (except Av4143) had more than 80% sensitivity. In contrast, only half (HF183, Rum-2-Bac, Pig-2-Bac, and Gull4) of rDNA-based assays reached this value. For markers targeted to bird feces, the use of the rRNA-based assay increased or at least did not change the performance. Regarding specificity, all the assays had >95% specificity with a DNA template, except the BacCan assay (71%). While using the RNA template for the assays, HF183 and BacCan exhibited only a low level of specificity (54 and 55%, respectively). Further, the HF183 assay amplified from multiple non-targeted animal fecal samples with the RNA template and the marker showed cross-amplification with the DNA template as well. This study recommends using the rRNA-based approach for MST assays targeting bird fecal contamination. In the case of mammal-specific MST assays, the use of the rRNA template increases the sensitivity but may reduce the specificity and accuracy of the assay. The finding of increased sensitivity calls for a further need to develop better rRNA-based approaches to reach the required assay performance.

## Introduction

Fecal contamination of surface water from human and animal sources causes a public health risk when the water is used for drinking or food production, but also recreational, such as swimming and diving (Soller et al., 2010; Kauppinen et al., 2019). In many cases, the discharges of non-disinfected municipal wastewater effluents are considered as the main sources of fecal pathogens in watersheds (Hokajärvi et al., 2013; Anza et al., 2014; Kauppinen et al., 2014), and also urban and agricultural runoffs are known to contain fecal pathogens (Uusi-Kämppä and Heinonen-Tanski, 2008; Rankinen et al., 2016). The most prevalent causes of waterborne infections in Finland, zoonotic fecal bacterial pathogen *Campylobacter* spp. and human-specific pathogenic noroviruses, are known to retain their pathogenicity in the cold conditions in water environments very well (Hörman et al., 2004; Hokajärvi et al., 2013; Kauppinen et al., 2014; Guzman-Herrador et al., 2015).

Fecal indicator bacteria (FIB), *Escherichia coli*, and intestinal enterococci are used for monitoring fecal contamination levels in surface waters. However, the current approach of monitoring FIB cannot differentiate the source of contamination. It assigns equal waterborne health risk levels for fecal contamination despite that the occurrence of pathogens is often source-dependent. For example, contamination from human and cattle sources in recreational water may cause a higher gastrointestinal illness risk for swimmers than the contamination from gull, chicken, or swine feces (Soller et al., 2010). In addition to needs from precise human health risk assessment, source differentiation between human, animal, or persisted environmental contamination is a prerequisite for mitigation of contamination sources, i.e., the causes of increasing FIB counts observed during regulatory monitoring (Sinigalliano et al., 2013; Tiwari et al., 2018). Animal-specific markers have been developed, for example, for swine, cattle, and birds, which are animal hosts known to carry zoonotic pathogens (Green et al., 2012; Ryu et al., 2012; Boehm et al., 2013). Over the recent decades, the DNA-based quantitative PCR (qPCR) of molecular MST markers of a variable region of the 16S rRNA gene of host-specific microbes have been developed and applied worldwide (Mieszkin et al., 2009; Haugland et al., 2010; Ryu et al., 2012; Boehm et al., 2013).

The environmental RNA can be changed into complementary DNA (cDNA) with the reverse transcriptase process and can be amplified with the same primers and probes, as done in the DNA-based method. Earlier studies reported that the rRNA-based method is more sensitive than the DNA-based method (Matsuda et al., 2012; Pitkänen et al., 2013; Kapoor et al., 2014). Our study hypothesizes that the high sensitivity of the rRNA assays may improve the MST efficiency in water samples even during a low level of fecal pollution. To our knowledge, the performance characteristics of such rRNA approaches have not been described before. Further, mitochondrial DNA (mtDNA)-targeted marker assays are an interesting option for MST due to their high host specificity (Caldwell and Levine, 2009; Malla and Haramoto, 2020). The mtDNA assays detecting epithelial cells defoliated from the intestinal tract of the hosts have been applied elsewhere for MST, but not tested before for environmental water samples in Finland.

Sensitivity, specificity, and accuracy of assays have been used for the characterization of the performance of different microbial methods (Boehm et al., 2013; Tiwari et al., 2018; Ballesté et al., 2020). Among such criteria, specificity is the primarily important character for any given host-specific MST assay. A false-positive MST assay result may lead to incorrect measures when the source tracking is utilized to reduce contamination of water areas or as a risk assessment tool (Tiwari et al., 2018). Ideally, MST markers should be highly specific to targeted hosts, and the markers should exist with high copy numbers in fecal materials to enable detection even after a dilution of fecal material in environmental waters. This study evaluates, for the first time, the performance characteristics of the rRNA-based template for MST. A collection of animal feces, sewage effluents, and surface water samples in different geographical locations of Finland were analyzed to determine the specificity, sensitivity, and accuracy of GenBac3 (general fecal contamination), HF183 (human), Rum-2-Bac (ruminant), Pig-2-Bac (swine), Gull4 (gull), GFD (birds), Av4143 (birds), DogND5 (dog), SheepCytB (sheep), and HorseCytB (horse) to be applied for use in MST investigations in watersheds with different levels of contamination.

## Materials and Methods

### Sampling Locations and Sampling

**FIGURE 1:**
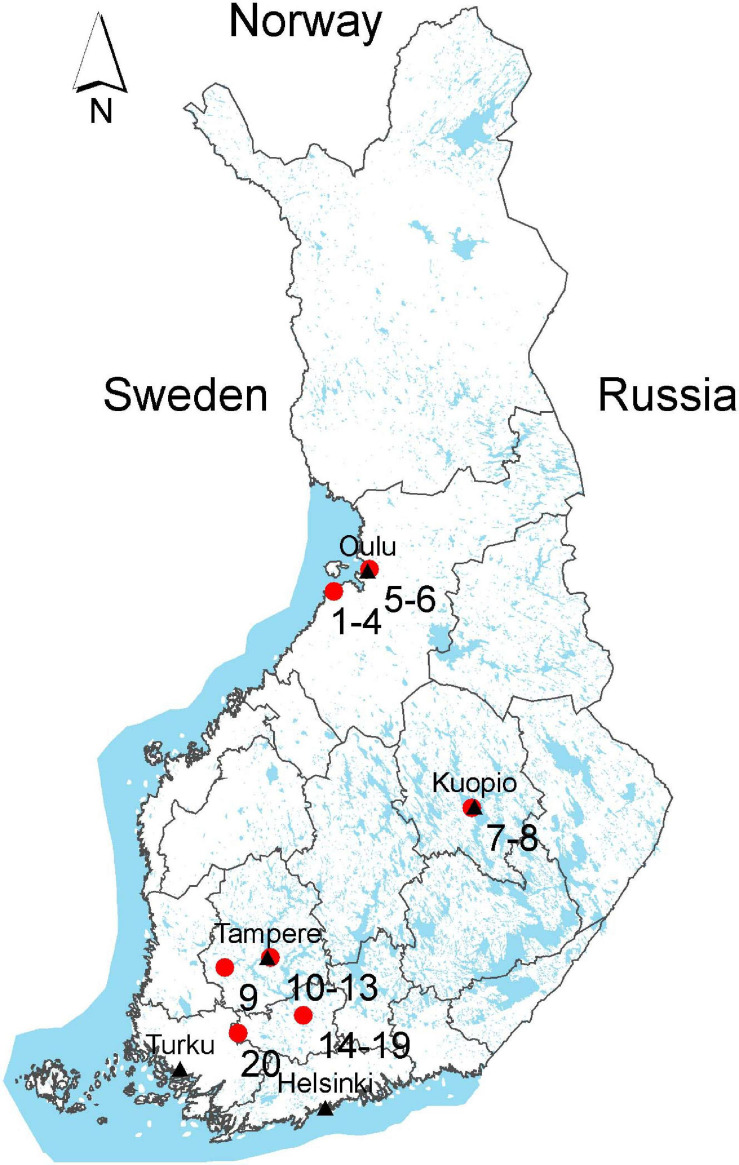
Water sample collection sites. Sites 1–6: Northern Ostrobothnia; sites 7–8: Northern Savonia; sites 9–13: Pirkanmaa; sites 14–20: Kanta-Häme (Map: National Land Survey of Finland; Sea area: Statistics Finland, Esri Finland). Each compartments on the map denotes 18 political regions of Finland, among them our study sampling covers four political regions.

Sample materials were collected between June and October in 2018 from six cities of four different geographical regions in Finland: Northern Ostrobothnia (sites 1–6), Northern Savonia (sites 7–8), Pirkanmaa (sites 9–13), and Kanta-Häme (sites 14–20) (Figure 1). A total of 95 surface water samples were collected from 33 water sampling sites, of which nine were from rural and 12 from urban areas, and five from public bathing areas (Supplementary Material 1 and Supplementary Table 1). Besides three sewage treatment plants, runoff water from two horse farms and a garden irrigation water site was sampled. In addition to the secondary (activated sludge) treated sewage effluent samples, waste water samples treated with LED-ultraviolet light (LED-UV, Led Future Inc., Kuopio, Finland) or exposed to wetland treatment were included (Uusheimo et al., 2018; Pitkänen et al., 2019). Half of the irrigation water samples were treated with LED-UV as well. Out of the 95 water samples, 85 were surface water and 10 were sewage effluent. Water samples of about 1 L were collected as grab samples into sterile plastic bottles.

Fecal samples for method development were collected nearby the water sampling sites within the same time frame. In addition, previously collected and stored gull and swine fecal material was used. Altogether, 151 fecal samples were used for method development (Table 1). The fecal samples were collected with a non-sterile disposable plastic spoon into a non-sterile re-sealable plastic bag.

**TABLE 1:** Summary of fecal samples collected and analyzed.

**Animal species**	**Sampling site**	**Sample count**	**Total**
Cattle (*Bos taurus*)	2	16	16
Sheep (*Ovis aries*)	2	19	19
Bird, unknown species	2	27	34
	6	1	
	14	6	
Horse (*Equus caballus*)	6	1	19
	8	2	
	Other, Northern Savonia region	16	
Dog (*Canis lupus familiaris*)	6	1	21
	14	20	
Goose (*Anserinae* spp.)	9	13	13
Duck (*Anatidae* spp.)	13	1	2
	14	1	
Waterfowl*	14	2	2
Gull (*Laurus* spp.)	Other, Northern Ostrobothnia, previously stored	17	17
Swine (*Sus scrofa domesticus*)	Other, previously stored	6	6
Hare (*Lepus europaeus*)	14	2	2
Total			151

*Unspecified species.

### Sample Transportation and Preservation

Samples were transported with sample coolers to the laboratory and processed within 24 h of sample collection. The water samples were filtrated onto 0.4-μm polycarbonate filters (as large volume as possible, 40–250 ml of effluents, and 50–600 ml of surface water) (Whatman Nuclepore Track-Etched Membranes, Sigma-Aldrich, United States). The membranes were frozen immediately after filtration and stored at −75°C or lower. A volume of 100 ml sterile-filtered water treated with diethyl pyrocarbonate (Invitrogen, Thermo Fisher Scientific, United States) was filtrated as negative filtration control. The fecal samples were distributed into 250-mg aliquots, frozen immediately, and stored at −75°C or lower.

### Nucleic Acid Extraction and Processing

The nucleic acids from the water samples were extracted using a Chemagic DNA Plant Kit (Perkin Elmer, United States) as previously described in Inkinen et al. (2019). An All Prep DNA/RNA Mini Kit (Qiagen, United States) was used for RNA and DNA extraction from fecal samples according to Pitkänen et al. (2013). Negative extraction controls with extraction reagents only and the negative filtration controls were processed alongside the samples. DNA concentrations were measured using Qubitds DNA HS assay kits and the Qubit 3.0 Fluorometer (Life Technologies, Thermo Fisher Scientific, United States). Immediately after the extraction, RNA aliquots were further purified using a TURBO DNA-free DNase kit, following the manufacturer’s instructions (Invitrogen, Thermo Fisher Scientific, United States). After purification, the RNA concentrations were measured using Qubit RNA HS assay kit and the Qubit 3.0 Fluorometer (Life Technologies, Thermo Fisher Scientific, United States). Following the extractions on the same day, the purified RNA was converted into complementary DNA (cDNA) by using the SuperScript IV VILO Master Mix system for RT-PCR, following the manufacturer’s instructions (Invitrogen, Thermo Fisher Scientific, United States) producing a total of 20 μl of each cDNA aliquot. To overcome the possible effect of reverse transcription inhibitors, the cDNA synthesis was performed using 8 μl as undiluted and 0.8 μl as 10-fold dilution of the total RNA. The total RNA was stored at −75°C or lower, while cDNA and DNA solutions were stored at −20°C until qPCR analysis.

### Quantitative Polymerase Chain Reaction

The performance of a total of 11 bacterial or mitochondrial marker assays (Table 2) was tested against the collection of fecal or wastewater samples, using qPCR assays with cDNA and DNA extracts as templates. The qPCR assays were performed using the QuantStudio 6 Flex real-time PCR system (Applied Biosystems, Thermo Fisher Scientific, United States). The TaqMan Environmental Master Mix 2.0 (Applied Biosystems, Thermo Fisher Scientific, United States) was used for TaqMan assays, and the Power SYBR Green PCR Master Mix (Applied Biosystems, Thermo Fisher Scientific, United States) was used for SYBR Green assays. Both DNA and cDNA were used as templates for most of the assays, but for assays targeting mtDNA, i.e., DogND5, HorseCytB, and SheepCytB assays, only a DNA template was used. The qPCR conditions are presented in Supplementary Table 2. The annealing temperatures described in the original assay publications (Table 2) were in accordance with the recommendations of the TaqMan and SYBR Green assay reaction mix manufacturers; thus, other cycling temperatures were not tested. The exception was optimization carried out for the assays GFD, HorseCytB, and SheepCytB, where a lower annealing temperature, 57°C, was considered. The performance characteristics (range of blanks, the limit of detection (LOD), amplification efficiency, *R*^2^-value, range of quantification, sensitivity, and specificity) remained indifferent between the tested annealing temperatures or were better with the higher annealing temperature of 60°C (Supplementary Table 2).

**TABLE 2:** The qPCR-assays used in the study.

**Target (assay)**	**Primer or probe sequence (5′ to 3′)**	**Chemistry**	**Annealing temp. (°C)**	**Size (bp)**	**References**
General *Bacteroidales* GenBac3	GenBactF3: GGGGTTCTGAGAGGAAGGT GenBactR4: CCGTCATCCTTCACGCTACT GenBactP2: 6-FAM-CAATATTCCTCACTGCTGCCTCCCGTA-ZEN/IBFQ	TaqMan	60	129	Siefring et al., 2008
Human-specific *Bacteroidalesdorei* HF183	HF183-1: ATCATGAGTTCACATGTCCG HfBthetR1: CGTAGGAGTTTGGACCGTGT HfBthetP1: 6-FAM-CTGAGAGGAAGGTCCCCCACATTGGA-ZEN/IBFQ	TaqMan	60	167	Haugland et al., 2010
Dog-specific *Bacteroidales* BacCan	BacCan-545f1: GGAGCGCAGACGGGTTTT BacUni-690r2: AATCGGAGTTCCTCGTGATATCTA BacUni-656p: 6-FAM-TGGTGTAGCGGTGAAA-BHQ1	TaqMan	60	145	Kildare et al., 2007
Ruminant-associated *Bacteroidales* Rum-2-Bac	RumBacB2-590F: ACAGCCCGCGATTGATACTGGTAA RumBac708Rm: CAATCGGAGTTCTTCGTGAT RumBacB2-626P: 6-FAM-ATGAGGTGGATGGAATTCGTGGTGT-ZEN/IBFQ	TaqMan	60	99	Mieszkin et al., 2010
Swine-specific *Bacteroidales* Pig-2-Bac	Pig-2-Bac41F: GCATGAATTTAGCTTGCTAAATTTGAT Pig-2-Bac163Rm: ACCTCATACGGTATTAATCCGC Pig-2Bac113MGB: 6-FAM-TCCACGGGATAGCC-MGB	TaqMan	60	116	Mieszkin et al., 2009
Gull-specific *Catellicoccus marimammalium* Gull4	qGull7F: CTTGCATCGACCTAAAGTTTTGAG qGull8R: GGTTCTCTGTATTATGCGGTATTAGCA qGull7P: 6-FAM-ACACGTGGGTAACCTGCCCATCAGA-ZEN/IBFQ	TaqMan	60	116	Ryu et al., 2012
Bird-specific *Helicobacter* spp. GFD	GFDF: TCGGCTGAGCACTCTAGGG GFDR: GCGTCTCTTTGTACATCCCA	SYBR Green	57	123	Green et al., 2012
Bird-specific *Lactobacillus* sp. Av4143	Av4143F: TGCAAGTCGAACGAGGATTTCT Av4143R: TCACCTTGGTAGGCCGTTACC Av4143P: 6-FAM-AGGTGGTTTTGCTATCGCTTT-BHQplus	TaqMan	60	244	Ohad et al., 2016
Dog mitochondrial gene NADH dehydrogenase subunit 5 DogND5	DogF: GGCATGCCTTTCCTTACAGGATTC DogR: GGGATGTGGCAACGAGTGTAATTATG DogP: 6-FAM-TCATCGAGTCCGCTAACACGTCGAAT-BHQ1	TaqMan	60	102	Caldwell and Levine, 2009
Sheep mitochondrial cytochrome B SheepCytB	SheepF: ACGCATTCATTGATCTCCCAGCTC SheepR: TCGGCAAATGTGGGTTACAGAGGA SheepP: 6-FAM-ACTTTGGCTCTCTCCTAGGCATTTGC-BHQ1	TaqMan	57	167*	Schill and Mathes, 2008
Horse mitochondrial cytochrome B HorseCytB	HorseF: AGGAGCAACAGTCATCACGAACCT HorseR: AAATGTACGACTACCAGGGCTGTG HorseP: 6-FAM-ATCGGTACTACCCTCGTCGAGTGAAT-BHQ1	TaqMan	57	168*	Schill and Mathes, 2008

6-FAM = 6-carboxyfluorescein. ZEN/IBFQ = ZEN-Iowa Black FQ quencher. BHQ1 = Black Hole Quencher 1. BHQplus = Black Hole Quencher plus. *The original article by Schill and Mathes (2008) did not define the amplicon size. The length was evaluated according to the standard sequences generated by NCBI Nucleotide BLAST.

The gBlocks Gene Fragments (Integrated DNA Technologies, United States), generated using reference sequences of the target sequences, selected by using the NCBI Nucleotide BLAST program (National Center for Biotechnology Information, United States National Library of Medicine), and including the exact primer and probe binding areas of the assays, were used for generating the standard curves. Ten-fold serial dilutions of these fragments were run with every assay with a total of 10 standard reactions per plate: 10^0^, 2 × 10^1^, 2 × 10^2^, 2 × 10^3^, 2 × 10^4^, and 10^5^ copies/μl. No template control (NTC) was run in duplicate with every standard set.

Undiluted and 1:10 and 1:100 diluted DNA and cDNA preparations in HyClone Water (GE Healthcare, Life Sciences, United Kingdom) were used to detect PCR inhibition. If inhibition was detected, the diluted samples were used for qPCR data generation. The limit of detection (LOD) was set as three copies per reaction, as suggested by Bustin et al. (2009). Background signals detected from negative extraction and filtration controls and LOD values were subtracted from all the results (clean NA) to generate the final data for the assay (Supplementary Table 3). Sample amount and dilution events from extraction, cDNA synthesis, and qPCR reaction (NA factor) were acknowledged. If the NA values were below the limit of quantification (LOQ), the result was treated as a present, but not quantitative, and therefore the value was set to half of the (0.5×) LOQ. The final data was calculated following the equations presented in Supplementary Material 2.

### Consecution of the Assay Performance Analysis

The genetic materials extracted and purified from the fecal samples of the selected host animals were amplified against the tested MST assays, with both RNA-based and DNA-based approaches. The amplification of the assay on targeted hosts was reported as true positive, and no amplification from the non-targeted hosts was considered as true negative. The amplification from the samples of non-targeted hosts was reported as a false-positive detection, and no amplification from the samples of the targeted host was reported as a false-negative result. The performance of the assays was evaluated by calculating sensitivity and specificity and accuracy with the following formulas 1–3 (Boehm et al., 2013; Tiwari et al., 2018; Ballesté et al., 2020):

(1) $$\text{Sensitivity}=\frac{T\,P\,\left(T\,r\,u\,e\,P\,o\,s\,i\,t\,i\,v\,e\right)}{T\,P+F\,N\,\left(F\,a\,l\,s\,e\,N\,e\,g\,a\,t\,i\,v\,e\right)}$$

(2) $$\text{Specificity}=\frac{T\,N\,\left(T\,r\,u\,e\,N\,e\,g\,a\,t\,i\,v\,e\right)}{T\,N+F\,P\,\left(F\,a\,l\,s\,e\,P\,o\,s\,i\,t\,i\,v\,e\right)}$$

(3) $$\text{Accuracy}=\frac{T\,P+T\,N}{T\,P+F\,P+T\,N+F\,N}$$

The sensitivity, specificity, and accuracy values of a marker of more than 80% are considered as reliable and acceptable (US Environmental Protection Agency, 2005). Further, the accuracy of the performance was predicted with the Bayes theorem as done earlier (Kildare et al., 2007) by using formula (4). As the prior probabilities were unknown in this case, the posterior probabilities were calculated by varying the prior probability from the worst-case scenario (negative signals in all samples or probability = 0) to the best-case scenario (positive signals in all samples or probability = 1) as described by Lamendella et al. (2009).

(4) $$P\,\left(A/B\right)=\frac{P\,\left(B/A\right)*P\,\left(A\right)}{P\,\left(B\right)}$$

In our case P(B) = P(B/A)* P(A) + P(B/A’)* P(A’), where

A = originated from a targeted host, B = test positive with the source. The probability of recording contamination from a certain source, when there is truly contamination, is defined by the following equation of the Bayesian theorem. *P(B/A)* is the test positive when there is contamination (True positive); *P(B)* is the total number of positive-tested cases that can be truly positive [P(B/A)] and false-positive [P(B/A’)].

### Statistical Analysis

All data above the LOD was logarithmic transformed (Log_10_) before further statistical analysis, as the original data did not follow a normal distribution. The statistical difference between copy numbers detected with RNA-based and DNA-based approaches was compared with the Mann–Whitney *U*-test. The differences in copy numbers on various hosts were compared with the Kruskal–Wallis test. The detection rate between RNA-based and DNA-based approaches was compared with the McNemar test. When the sample number was less than 20, Fisher’s exact test was used to confirm the result. The difference was considered statistically significant when *p* < 0.05. All the statistical tests were conducted in IBM (2020), and figures were made on Origin (Pro), 2017.

## Results

### Performance of qPCR Amplification

The qPCR assay characteristics are summarized based on the amplification of the targeted assay on negative control, LOD, amplification efficiency, *R*^2^-value of the amplification curve, and the range of quantification from the qPCR runs with fecal samples in Supplementary Table 3. Except for Av4143, the lower range of amplification efficiency of the assays was above 80%. The *R*^2^-value of the amplification curve ranged between 0.946 and 1.000; the highest was with a SheepCytB marker, and the lowest was in the Av4143 marker. All the assays had the range of quantification from 10 to 10^5^ GC per μl template, except BacCan, which had the range of 10^2^ to 10^5^ GC per μl template. GenBac3 and DogND5 assays with a DNA-based method, and GenBac3, GFD, and Rum2-Bac assays with an RNA-based method, showed some amplification in the blank samples (Supplementary Table 3).

### Copy Numbers and Detection Frequency on Fecal Samples

**FIGURE 2:**
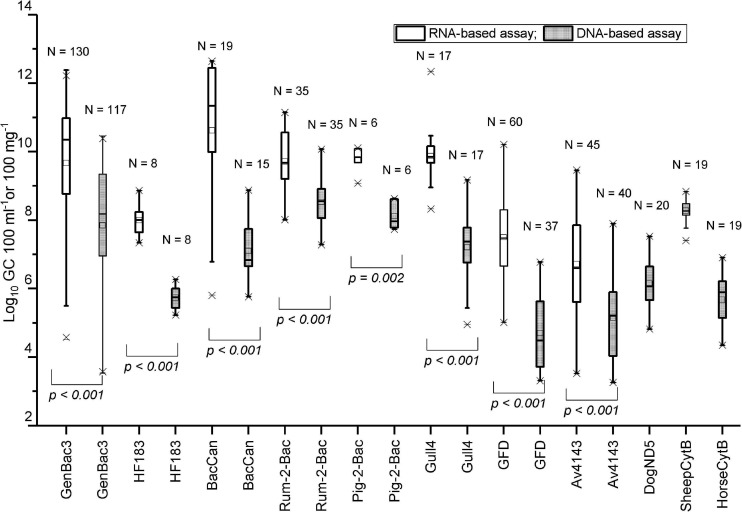
The comparison between RNA-based and DNA-based copy numbers. The fecal and sewage samples were analyzed using the respective host-specific MST marker assays. The *p*-Value is based on the Mann–Whitney *U*-test. N: the number of samples included in the comparison (>LOQ).

All bacterial assays produced higher copy numbers with the RNA-based approach than the DNA-based approach (*p* < 0.001–0.002, Mann–Whitney *U*-test; Figure 2). On average, BacCan assay produced the highest (10.6 log_10_) and the Av4143 assay produced the lowest (6.7 log_10_) RNA copy numbers per 100-mg feces, and the Rum-2-Bac assay produced the highest (8.5 log_10_) and GFD assay produced the lowest (4.7 log_10_) DNA copy number per 100-mg feces of targeted hosts (Figure 2 and Supplementary Table 4).

Regarding detection frequency, the microbial targets were more frequently or at least equally detected from fecal samples with the RNA-based approach than with a DNA-based approach (Supplementary Table 4). The GFD assay was more frequently detected from targeted fecal samples with the RNA-based approach than with the DNA-based approach (p = 0.002, McNemar test, Supplementary Table 4). Assays HF183, Rum-2-Bac, Pig-2-Bac, and Gull4 had a 100% detection rate with both RNA-based and DNA-based approaches. Assays GenBac3, BacCan, and Av4143 had a higher detection percentage rate with the RNA-based approach, but the difference was not significant (Supplementary Table 4). Mostly, the detection frequency of assays in non-targeted fecal or sewage samples was increased with the RNA-based approach in comparison with the DNA-based approaches (Table 3).

**TABLE 3:** Sample-specific results of fecal samples.

**Fecal source**	**Microbial source tracking markers**																																					
	**GenBac3**				**HF183**				**BacCan**				**Rum-2-Bac**				**Pig-2-Bac**				**Gull4**				**GFD**				**Av4143**				**Dog ND5**		**HorseCytB**		**SheepCytB**	
	**RNA**		**DNA**		**RNA**		**DNA**		**RNA**		**DNA**		**RNA**		**DNA**		**RNA**		**DNA**		**RNA**		**DNA**		**RNA**		**DNA**		**RNA**		**DNA**		**DNA**		**DNA**		**DNA**	
	**T**	**NT**	**T**	**NT**	**T**	**NT**	**T**	**NT**	**T**	**NT**	**T**	**NT**	**T**	**NT**	**T**	**NT**	**T**	**NT**	**T**	**NT**	**T**	**NT**	**T**	**NT**	**T**	**NT**	**T**	**NT**	**T**	**NT**	**T**	**NT**	**T**	**NT**	**T**	**NT**	**T**	**NT**
Effluent (*n* = 8)	8	−	8	−	8^#^	−	8^#^	−	nc	nc	nc	nc	nc	nc	nc	nc	nc	nc	nc	nc	nc	nc	nc	nc	nc	nc	nc	nc	nc	nc	nc	nc	nc	nc	nc	nc	nc	nc
Dog (*n* = 21)	21	−	21	−	−	11	−	1	19	−	16	−	−	3	−	−	−	−	−	−	−	−	−	1	−	1	−	−	−	3	−	2	20	−	−	−	−	1
Cattle (*n* = 16)	16	−	16	−	−	9	−	−	−	16	−	12	16	−	16	−	−	−	−	−	−	−	−	−	−	−	−	−	−	−	−	−	−	−	−	−	−	−
Swine (*n* = 6)	6	−	6	−	−	6	−	−	−	6	−	3	−	−	−	−	6	−	6	−	1	−	−	−	−	−	−	−	−	−	−	−	−	−	−	−	−	−
Sheep (*n* = 19)	19	−	19	−	−	11	−	3	−	19	−	19	19	−	19	−	−	−	−	−	−	1	−	−	−	−	−	−	−	−	−	−	−	−	−	−	19	−
Horse (*n* = 19)	19	−	19	−	−	14	−	−	−	10	−	2	−	−	−	1	−	−	−	−	−	−	−	−	−	−	−	−	−	−	−	−	−	−	19	−	−	−
Hare (*n* = 2)	2	−	2	−	−	2	−	2	−	1	−	−	−	−	−	−	−	−	−	−	−	−	−	−	−	−	−	−	−	−	−	−	−	−	−	−	−	−
Gull (*n* = 17)	13	−	8	−	−	15	−	1	−	−	−	−	−	−	−	−	−	2	−	−	17	−	17	−	15	−	8	−	16	−	14	−	−	−	−	−	−	−
Goose (*n* = 13)	11	−	9	−	−	2	−	−	−	4	−	1	−	−	−	−	−	−	−	−	−	−	−	2	13	−	8	−	1		1	−	−	−	−	−	−	−
Duck (*n* = 2)	1	−	1	−	−	−	−	−	−	−	−	−	−	−	−	−	−	−	−	−	−	−	−	−	1	−	1	−	1		1	−	−	−	−	−	−	−
Waterfowl (*n* = 2)	2	−	1	−	−	−	−	−	−	1	−	−	−	−	−	−	−	−	−	−	−	−	−	−	2	−	2	−	2		2	−	−	−	−	−	−	−
uBird (*n* = 34)	9	−	11	−	−	−	−	−	−	2	−	1	−	2	−	1	−	−	−	−	28	−	26	−	29	−	26	−	25	−	25	−	−	−	−	−	−	−

*The different animal species and sewage effluent were analyzed using the tested MST assays with RNA-based and DNA-based templates. ^#^Samples before wetland treatment, T, targeted; NT, not targeted; nc, not considered; −, none; uBird, fecal samples from birds with unidentified species.*

### Host Specificity and Cross-Reactivity With RNA-Based and DNA-Based Approaches

The GenBac3 assay targeting general Bacteroidales has been detected in 100% of mammal fecal samples with both RNA-based and DNA-based approaches but relatively less frequently (57% with RNA and 49% with DNA) from bird feces (Table 3, *p* < 0.001, McNemar test). There were also significantly higher GenBac3 copy numbers in the fecal material of mammals than in the fecal materials of birds, with both RNA-based and DNA-based approaches (Supplementary Figure 1; *p* < 0.001, Mann–Whitney *U*-test). However, there was no significant difference in the distribution of GenBac3 DNA (*p* = 0.177, Kruskal–Wallis) or the RNA (*p* = 0.199, Kruskal–Wallis) marker between bird (unknown bird species, gull, and goose) feces, but the distribution of the GenBac3 DNA marker was significantly different between mammal feces (horse, cow, sheep, dog, swine; *p* < 0.001, Kruskal–Wallis) (Supplementary Figure 1). Specifically, dog and cow feces expressed significantly lower GC numbers with the GenBac3 DNA-based approach than horse, swine, and sheep feces. Horse feces expressed significantly lower GC numbers with a GenBac3 DNA-based approach than sheep feces. As well, the distribution of the GenBac3 RNA marker was significantly different between mammal feces (horse, cow, sheep, dog, swine; *p* < 0.001, Kruskal–Wallis) (Supplementary Figure 1). Specifically, sheep feces expressed significantly lower GC numbers with the GenBac3 RNA-based approach than cow and dog feces. Horse feces expressed significantly lower GC numbers with the GenBac3 RNA-based approach than dog feces. The overall sensitivity of the GenBac3 assay was 81% with RNA-based and 77% with DNA-based templates (Table 4). The sample material in this study did not include true negative (non-fecal) samples for the GenBac3 assay; therefore, the specificity and accuracy of this assay were not calculated.

**TABLE 4:** Performance characteristics of the MST assays when using RNA and DNA as a template.

**Assay**	**Sensitivity (%)**		**Specificity (%)**		**Accuracy (%)**	
	**RNA**	**DNA**	**RNA**	**DNA**	**RNA**	**DNA**
GenBac3^#^	81	77	NA	NA	NA	NA
HF183^##^	100	100	54	95	56	96
BacCan	90	76	55	71	60	72
Rum-2-Bac	100	100	96	98	97	99
Pig-2-Bac	100	100	99	100	99	100
Gull4	100	100	97	97	97	97
GFD	88	66	99	100	94	85
Av4143	66	57	96	97	83	79
DogND5	NA	95	NA	100	NA	99
SheepCytB	NA	100	NA	99	NA	99
HorseCytB	NA	100	NA	100	NA	100

*^#^The specificity and accuracy were not evaluated since the sample material did not include true negative samples for the GenBac3 assay. ^##^HF183 assay values were determined by using sewage effluent samples before wetland treatment. NA, not applicable.*

**FIGURE 3:**
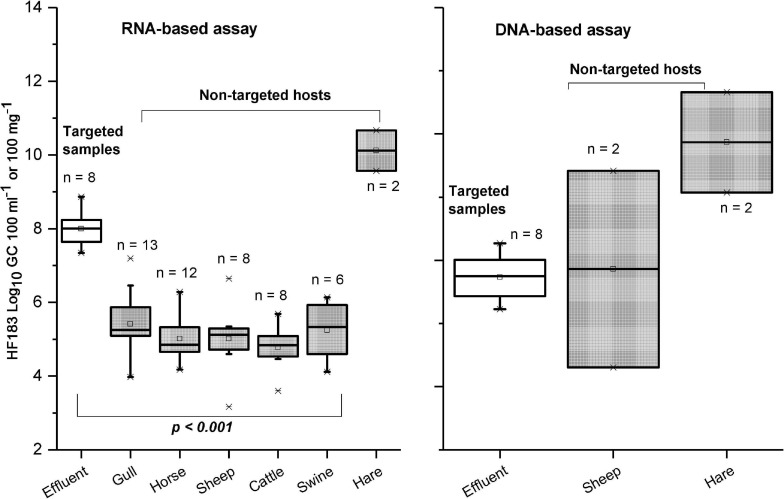
Copy numbers generated with HF183 assay for targeted (sewage) and non-targeted (animal fecal) samples when using RNA and DNA as a template. Only the samples with results > LOQ included. The *p*-Value is based on the Kruskal–Wallis test.

The HF183 marker was detected in all targeted samples (sewage effluents before efficient tertiary treatment) from both RNA-based and DNA-based templates (Table 3 and Supplementary Table 4). However, cross-reactions with non-targeted animal species (dog, cattle, swine, sheep, horse, hare, gull, and goose) happened more frequently with the RNA-based approach, compared with the DNA-based approach, which cross-reacted dog, sheep, hare, and gull (Table 3). The copy numbers from the RNA-based approach were significantly higher in targeted fecal samples than in non-targeted samples (Figure 3). The only exception was from the two hare fecal samples, from which the HF183 assay resulted in the highest recorded copy numbers from both RNA and DNA templates (Figure 3). The statistical test was not possible for DNA-based results due to a low number of samples exhibiting false-positive signals. The sensitivity of the HF183 assay was 100% with both RNA-based and DNA-based assays, but specificity was much lower, being 54% when RNA was the template and 95% when the template was DNA (Table 4). The accuracy of the HF183 assay was 56% with the RNA-based and 96% with the DNA-based approach.

**FIGURE 4:**
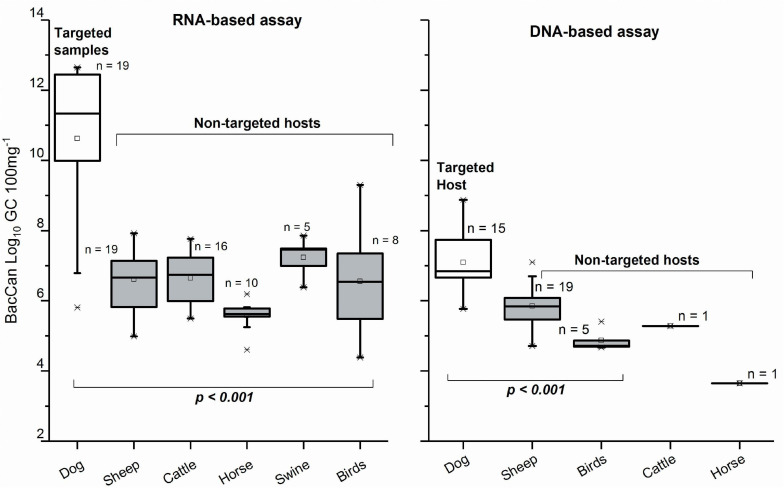
Copy numbers generated with BacCan assay for targeted (dog) and non-targeted animal fecal samples when using RNA and DNA as a template. Only the samples with results > LOQ included. The *p*-Values are based on the Kruskal–Wallis test.

From a total of 21 dog fecal samples, the dog-specific BacCan assay showed amplification in 19 with the RNA-based and 16 with the DNA-based approach. The sensitivity of the BacCan assay was 90% in the RNA-based and 76% in the DNA-based approach. The usability of this marker was questioned as the marker was amplified from fecal samples of mostly all animal species sampled in this study with both RNA-based and DNA-based approaches (Table 3). However, the BacCan copy numbers were significantly lower in the feces of non-targeted hosts as compared to the targeted canine feces (Figure 4). The specificity of the BacCan assay was 55% with an RNA-based and 71% with a DNA-based approach (Table 4). Further, the accuracy of this assay was 60% with RNA-based and 70% with RNA-based and DNA-based approaches.

The ruminant-specific Rum-2-Bac marker was 100% sensitive with cattle and sheep fecal samples with both RNA-based and DNA-based templates (Table 4). However, the detected GC was significantly higher in cattle fecal samples compared to sheep fecal samples (*p* < 0.001, Mann–Whitney *U*-test, Supplementary Table 5). The Rum-2-Bac marker cross-reacted with three dog and two bird fecal samples with the RNA-based approach and with one horse and one bird fecal sample with the DNA-based approach (Table 3). The marker had 96% specificity and 97% accuracy with the RNA-based approach and 98% specificity and 99% accuracy with the DNA-based approach.

Among bird-specific markers, the gull marker Gull4 was 100% sensitive with gull feces with both RNA-based and DNA-based assays (Table 4). There was a significant difference between the detected GC in the gull fecal samples compared to the fecal samples from unknown bird species with the RNA-based approach (*p* < 0.001, Kruskal–Wallis test), but the difference detected with the DNA-based approach was no longer significant (*p* = 0.518, Mann–Whitney *U*-test, Supplementary Table 5). The marker cross-reacted with one sheep fecal sample with the RNA-based approach and two goose and one dog fecal samples with the DNA-based approach (Table 3). The specificity and accuracy of this marker were 97% for both RNA-based and DNA-based approaches (Table 4). The sensitivity of the bird markers GFD and Av4143 were low, varying from 57 to 88% (Table 4). Instead, the specificity of the GFD assay was 99% with an RNA-based and 100% with a DNA-based approach, and specificity of the Av4143 assay was 96% with RNA-based and 97% with DNA-based approaches. Bird markers GFD and Av4143 cross-reacted with only a few (<3) canine fecal samples (Table 3). There were no significant differences in GC numbers between the different studied bird species (Supplementary Table 5).

In comparison to host-specific MST assays targeted to the 16S rRNA of bacteria, the performance of the mtDNA-based assays was generally better in terms of sensitivity, specificity, and accuracy calculated from the fecal sample test results (Table 4). The dog-specific assay DogND5 had the lowest (95%) sensitivity, whereas sheep-specific assay SheepCytB was the only one cross-reacting with one non-target (dog) sample (Table 3).

### Probability of Target Detection

**FIGURE 5:**
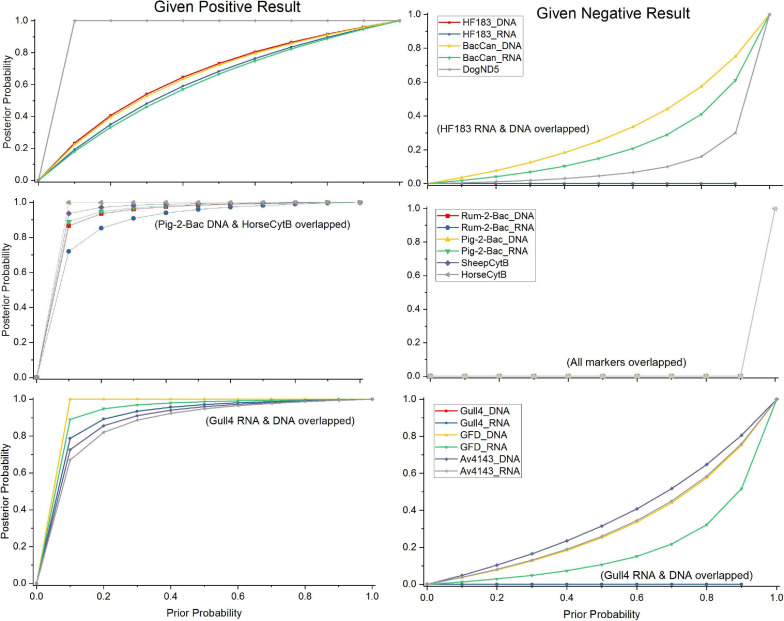
The probabilities of true-positive and false-negative results in the case of animal fecal contamination by the Bayesian statistical model. Posterior probabilities of contamination given a positive qPCR result using the markers specific for dog, sheep, and bird, as well as human, gull, horse, and swine over a range of prior probabilities.

The probability of the tested MST markers, except the general fecal marker GenBac3, to correctly detect the presence of their targeted host feces in the water when using RNA and DNA as a template, was studied by the Bayesian statistical model. For the host-specific assays, as the prior probabilities were unknown, the range of prior probabilities from the worst-case scenario to the best-case scenario was used to visualize the performance of the markers to correctly detect their target. When the markers were compared by their ability to produce a positive result, in case the water matrix was contaminated with the feces of the target animal (Figure 5), the DogND5 assay exhibited a better capacity to correctly assign canine fecal contamination than the BacCan assay. Further, the capacity of the SheepCytB assay for detecting sheep feces, and the GFD assay for detecting bird feces was better than the capacity of the Rum-2-Bac and Av4143 assays, respectively. Nevertheless, the Rum-2-Bac and Av4143, as well as the HorseCytB, Pig-2-Bac, and Gull4 markers, showed a relatively good capacity to correctly detect their targets. By contrast, the capacity for the correct detection of BacCan and HF183 was relatively weak.

### Detection of MST Markers in the Surface Water Samples

**FIGURE 6:**
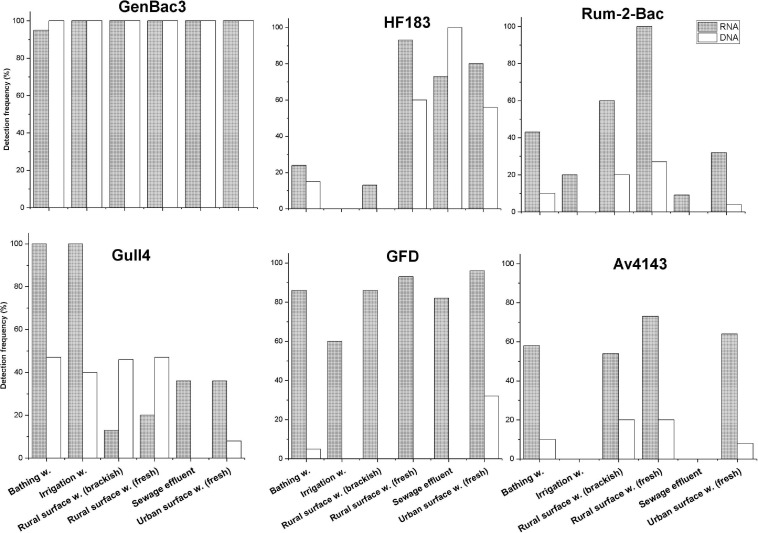
Detection frequency (%) of the MST markers in the groups of surface water samples with both RNA-based and DNA-based approaches. Only markers with detection frequency > 20% in surface water groups are shown. In *x*-axis: sample group. W, water.

All bacterial markers (Rum-2-Bac, Gull4, GFD, and Av4143) were more frequently detected with the RNA-based approach than the DNA-based approach from the majority of the surface water sample types (*p* < 0.001, McNemar test, Figure 6), as well as all the samples together (Supplementary Table 6). In most of the sample groups, the copy number of rRNA was significantly higher than the rDNA copy number with all markers (*p* < 0.001, Mann-Whitney *U*-test, Supplementary Figures 2–7).

The RNA-based GenBac3 marker was detected in all sample groups in 98% of the total samples, and DNA-based GenBac3 markers were detected in all (100%) samples (Figure 6). The highest median of GC of 9.9 log_10_ GC/100 ml with an RNA-based approach and 7.07 log_10_ GC/100 ml with a DNA-based approach was detected in sewage samples (Supplementary Figure 2). Bathing water samples had the lowest GC values with both RNA-based (6.68 log_10_ GC/100 ml) and DNA-based (4.12 log_10_ GC/100 ml) approaches (Supplementary Figure 2).

The HF183 marker was detected from all sewage samples (100%) with a DNA-based approach. When an RNA-based approach was employed, the HF183 marker was detected more frequently from rural (fresh) surface water samples (93%, Figure 6). The RNA-based marker was detected only in 73% of sewage effluent samples, which was a lower detection rate than detected from urban surface water samples (80%). However, the highest median GC values were detected in sewage samples by using both RNA-based (7.73 log_10_ GC /100 ml) and DNA-based (5.45 log_10_ GC /100 ml) templates (Supplementary Figure 3). The detection frequency of this marker was less than 20% in bathing water, irrigation water, and rural (brackish) surface water samples.

The Rum-2-Bac marker was more frequently detected in rural brackish (60%) and fresh (100%) surface water when an RNA-based template was used. Overall, the detection frequency of the Rum-2-Bac marker was low when a DNA-based template was used. The highest detection frequency for a DNA-based template was 27% in rural (fresh) surface water samples. The highest median value of an RNA-based Rum-2-Bac assay was also detected from rural fresh surface water (5.38 log_10_ GC/100 ml) (Supplementary Figure 4). The Pig-2-Bac marker was not detected from any surface water samples in this study.

The Gull4 RNA markers were detected from all bathing water and irrigation water samples. However, the Gull4 DNA marker was detected in only about 50% of rural surface water (fresh and brackish), bathing water, and irrigation water samples (Figure 6). The highest median GC value with an RNA-based approach (5.89 log_10_ GC/100 ml) was detected in bathing water samples (Supplementary Figure 5).

The general bird-specific marker GFD was detected with the RNA-based approach in all sample groups. The highest detection frequency (96%) was noted in urban surface water and the lowest (60%) in irrigation water. By using the DNA-based approach, the GFD marker was detected only in urban surface water (32%) and bathing water (5%). The highest median value with RNA-based template (5.32 log_10_ GC/100 ml) was also from the urban surface water sample (Supplementary Figure 6). The other bird-specific marker Av4143 was less frequently detected in comparison to the corresponding GFD detection frequency when an RNA-based approach was used. On the contrary, Av4143 was detected more frequently than GFD when the DNA-based approach was used. The RNA-based Av4143 marker was not detected from sewage and irrigation water samples, although it was detected in 54% of rural surface water and 73% of urban surface water samples. The highest detection frequency with a DNA-based approach was only 20%, and it was reached from rural surface water (fresh and brackish). The highest median value (4.48 log_10_ GC/100 ml) of Av4143 RNA was also from the urban surface water sample (Supplementary Figure 7).

Mitochondrial DNA markers were less frequently detected in surface water samples than bacterial markers. A DogND5 marker was detected from two bathing water samples and one urban surface water sample with GC ≤ 3.43 log_10_ GC/100 mL. HorseCytB was detected only from one out of three samples from horse-farm runoff, and it was not detected at all from other environmental samples. SheepCytB was not detected from the environmental samples tested in this study.

## Discussion

This study tested the performance of previously reported MST assays targeting general fecal contamination, human, gull, ruminant, swine, dog, horse, sheep, and general birds. The goal was to utilize the cDNA template produced from RNA, with a reverse transcriptase process, and compare its performance with a currently used rDNA-based approach and mtDNA-based approaches. To our knowledge, this is the first study to evaluate the performance of MST assays in Finland. Furthermore, to our knowledge, this is the first study to evaluate the performance of 16S rRNA-targeted MST assays with the use of the RNA-based template instead of the DNA template. As a main finding, the sensitivity, specificity, and accuracy of the assays targeting bird feces (Gull4, GFD, and Av4143) measured with the RNA-based approach were higher than, or at least similar to, the conventional DNA-based approach. In the case of mammal-specific markers (HF183, Rum-2-Bac, and Pig-2-Bac), the RNA-based approach resulted in a higher sensitivity, but the assay specificity and accuracy were lower than when using the DNA-based template.

A microbial assay with higher counts (CFU or GC) in fecal material has greater significance for water-quality monitoring; for example, such assay remains still detectable even after many folds of dilution in a surface water resource (Harwood et al., 2009, 2014; Layton et al., 2013). Sensitivity refers to the proportion of known positive controls that are correctly identified as positive. The higher sensitivity has practical significance; it better protects the public health than the methods with lower sensitivity (Harwood et al., 2014).

Laboratory methodologies developed in one geographical region mostly have global applicability. However, in the case of MST assays, mainly targeting host-specific bacteria, the assay performance can vary between the geographical locations, as gut bacterial communities are affected by animal feeding practices, herd size, and ages (Dick et al., 2005; Shanks et al., 2011; Ballesté et al., 2020). Such possible variation requires verification of the accuracy and reliability of MST markers before using them in a new geographical location (Roslev and Bukh, 2011). However, the probable fecal contamination sources in each watershed are different and each earlier study tested the marker in different animal fecal materials. In fact, multiple previous studies reported the cross-reaction of MST markers with the fecal materials from non-targeted species (Ryu et al., 2012; Boehm et al., 2013; Sinigalliano et al., 2013). Therefore, the performance characteristics, mainly specificity, related to the false-positive rate of the assays should be carefully evaluated in a new geographical location (Harwood et al., 2009; Stewart et al., 2013).

### Comparing the Performance of the RNA-Based and DNA-Based Approaches

As reported in earlier studies (Pitkänen et al., 2013; Kapoor et al., 2014), also in our study, the RNA-based assays targeted to 16S rRNA had a higher detection frequency and higher target copy numbers in fecal samples and also in surface water samples than the DNA-based assays. The explanation for the increased sensitivity is that an active cell contains ribosomes full of ribosomal RNA (Waters and McCuthan, 1990; Nogales et al., 2001; Péìrez-Osorio et al., 2010). The rRNA target may indicate the activity and transcription rate status of bacterial cells, as metabolically active cells have greater amounts of rRNA per cell than non-viable cells (Gourse et al., 1996; Martinez et al., 2006; Péìrez-Osorio et al., 2010).

In line with earlier studies using DNA as a template (Mieszkin et al., 2009, 2010; Ryu et al., 2012; Boehm et al., 2013; Raith et al., 2013; Harwood et al., 2014; Ohad et al., 2016), the sensitivity of HF183, Gull4, Rum-2-Bac, and Pig-2-Bac assays was more than 80% also in this study. In this study, this sensitivity was reached with both RNA-based and DNA-based approaches. Instead, the sensitivity of BacCan, GenBac3, and GFD assays remained below 80% when DNA was used as a template, while others have reported sensitivities of 63–100%, 100%, and 30–68%, respectively (Kildare et al., 2007; Boehm et al., 2013; Ahmed et al., 2015, Ahmed et al., 2016; Odagiri et al., 2015; Nshimyimana et al., 2017; Symonds et al., 2017). More than 80% sensitivity was achieved when RNA was used as a template. Of these assays, the GenBac3 assay was poorly amplified in the fecal materials of birds, with a lower detection rate and lower GC in comparison with mammal fecal materials. Earlier studies also reported the variation in the proportions of *Bacteroidetes* between different groups of birds, almost absent in waterfowls and broiler chickens, present in battery hens, and dominant in turkeys (Zhu et al., 2002; Scupham et al., 2008; Lu et al., 2009). However, such differences between the studied bird species were not noticed in this study.

The performance characteristics sensitivity, specificity, and accuracy of the human-specific marker (HF183) were good, being higher than 95% when a DNA-based template was used in this study. Surprisingly, the specificity and accuracy of this assay dropped to the levels of 54% and 56%, respectively, when we used rRNA as a template for the HF183 assay. Also, earlier studies with a DNA-based approach have reported cross-amplification of the HF183 assay with non-targeted species, such as dog, rabbit, chicken, swine, and cattle, with specificities between 80 and 100% (Boehm et al., 2013; Layton et al., 2013; Odagiri et al., 2015; Nshimyimana et al., 2017; Haramoto and Osada, 2018). Although the specificity of the RNA-based approach was lower than the specificity of the DNA-based approach in the present study, the GC difference in the fecal material between targeted and non-targeted hosts was many folds higher with the RNA-based approach than in the DNA-based approach. Thus, the cases where HF183 was detected in urban surface water simultaneously with both RNA-based and DNA-based approaches could tentatively be explained by human-derived fecal contamination such as accidental leakages of municipal sewage. However, the high cross-reactivity of the HF183 marker on a non-targeted host feces, especially with an RNA-based approach, calls for a need for method development toward more specific but still enough sensitive markers for human fecal contamination. For example, totally new targets could be found from the rapidly increasing metagenome data and also the further optimization of the PCR conditions of the current genetic targets might improve the assay performance as well.

In the case of the BacCan marker, many earlier studies have also reported the poor performance characteristics (Kildare et al., 2007; Boehm et al., 2013; Schriewer et al., 2013; Odagiri et al., 2015; Nshimyimana et al., 2017). For example, Schriewer et al. (2013) reported 100% sensitivity, but only 70% specificity of the BacCan assay. In our hands, the BacCan assay cross-reacted with feces of nearly all studied animal species. It is noteworthy that the GC counts were many folds lower in the feces of non-targeted hosts than the targeted hosts. This difference in the copy numbers was even greater when using rRNA as a template for the assay. However, acknowledging the poor specificity of this marker, it was discarded before the water sample analysis. Herein, the ruminant-specific Rum-2-Bac and Pig-2-Bac had similar performance characteristics with earlier studies (Mieszkin et al., 2009, 2010; Boehm et al., 2013; Raith et al., 2013). The highest detection rate of the Rum-2-Bac assay (RNA: 100% and DNA: 27%) fresh rural surface water confirms our hypothesis: the sensitivity of the RNA-based approach is crucial for the contamination source detection from watersheds. Regarding swine-specific Pig-2-Bac, Mieszkin et al. (2009) reported 98–100% sensitivity and 100% specificity while testing fecal materials from pig, cow, sheep, and horse. Haramoto and Osada (2018) reported 100% sensitivity, 66% specificity, and 77% accuracy of the Pig-2-Bac assay with a DNA-based assay. They reported the Pig-2-Bac marker amplification on cattle feces.

The performance of the Gull4 marker with the RNA-based and DNA-based approaches was in line with earlier studies (Ryu et al., 2012; Ohad et al., 2016; Ballesté et al., 2020). Ballesté et al. (2020) reported 85% sensitivity and 100% specificity of the Gull4 marker while testing the assay in human, ruminant, sheep, horse, pig, and gull feces. Ryu et al. (2012) reported 87% sensitivity and 91% specificity of the Gull4 assay with the DNA-based approach. The Gull4 marker had the highest detection frequency in irrigation water and bathing water (100% for both) among the six different surface water sample types studied (Figure 6). As already noted with other assays, the sensitivity of the GFD assay was also higher (88%) with the RNA-based approach than with the DNA-based approach (66%). In comparison, Green et al. (2012) reported sensitivity of 58%, whereas Ahmed et al. (2016) reposted sensitivities of 58% from a Brisbane, Australia sample and 30% from a Florida, United States sample (52% when combined). Symonds et al. (2017) reported 44% sensitivity (chicken and sea birds) and 56% specificity (cross-amplified with cow, dog, sewage, horse, and pig fecal materials) of this assay with a DNA-based approach. Therefore, the use of rRNA as a template seems to bring a needed boost for the assay sensitivity. The use of rRNA as a template seems feasible with the GFD assay, as the specificity of the assay was as good as 99% with an RNA-based approach (it was 100% when rDNA was used as a template). In the performance evaluation, the GFD assay was sensitive for the fecal material of multiple birds, including gulls, ducks, goose, and waterfowl, and when analyzing fecal samples from unspecified bird species. The GFD marker was the most frequently detected in urban surface water (96%) and sewage effluent (82%), among six studied sample groups (Figure 6) with an RNA-based approach. This study recorded low sensitivity for the other bird marker tested, the Av4143 assay (RNA 66%, DNA 57%). This result deviates from an earlier study, where Ohad et al. (2016) reported a 95% sensitivity for this assay. The poor sensitivity of the Av4143 marker on different bird fecal materials may indicate that the bacterial group targeted with this marker may not be present in the gut of all bird populations. The potential geographical instability of this marker calls for further investigation. Due to the study outcome, we recommend the use of the GFD assay instead of Av4143 for use in Finnish surface water quality monitoring.

Although in general, the number of fecal samples per host used for this performance analysis was large, the HF183 and Pig-2-Bac assays targeting human and swine fecal contamination, respectively, were evaluated by using only eight sewage effluent and six swine fecal samples, which is below the recommended size of ten samples per each targeted host (US Environmental Protection Agency, 2005).

### Mitochondrial DNA-Based Assays

This study demonstrated excellent performance characteristics of SheepCytB and HorseCytB assays. The high sensitivity and specificity (95–100%) of these mtDNA-based assays were consistent with earlier findings (Caldwell and Levine, 2009; Tambalo et al., 2012; He et al., 2016; Malla and Haramoto, 2020). The dog-specific DogND5 assay had higher sensitivity, specificity, and accuracy than the respective BacCan assay targeted to host-specific fecal bacteria. The central assumption of the host-specific, bacteria-based approach is that the targeted bacteria (or groups) have a strong relationship with a particular host. However, these fecal bacteria can be found from the feces of non-targeted hosts too, as noted in this study. In contrast, in the mtDNA-based MST approach, the detection of target DNA from exfoliated epithelial cells from the host alimentary canal has a much higher specificity than bacterial assays (Caldwell et al., 2011; Malla and Haramoto, 2020).

The usefulness of the highly specific mtDNA-based assays is somewhat hampered with the fact that the amplification efficiency of the assays could be relatively weak, as noted when using DogND5, SheepCytB, and HorseCytB assays for water sample testing in this study. The exact reason for low efficiency in our hands remains unclear, and some earlier studies have reported the higher amplification efficiency values of the DogND5 assay (Caldwell and Levine, 2009; Tambalo et al., 2012). Although the sensitivity and specificity of all three mtDNA-based assays were outstanding (∼100%) when fecal materials were tested, these targets were only seldom detected from the surface water samples. The mtDNA assays remained negative even when the feces of the targeted host animals were suspected to be present in the water, which creates uncertainty for the applicability of the mtDNA assays in real life. The dog-specific DogND5 marker was not detected from fresh rural and brackish rural surface water, where the detection was noted by the BacCan bacterial assay (RNA-based approach). However, due to the obvious specificity issues with RNA-based BacCan detection, the absence of the target feces from the samples tested cannot be out ruled either.

## Conclusion

• The performance characteristics sensitivity, specificity, and accuracy of assays targeting birds with an RNA-based approach were higher than or equal to the DNA-based approach.
• The sensitivity of human and dog markers were higher with the RNA-based approach, but specificity and accuracy were higher with the DNA-based approach. The performance between using RNA and DNA as a template was similar to ruminant and swine markers.
• The performance of assays DogND5, HorseCytB, SheepCytB, GFD, Gull4, Rum-2-Bac, and Pig-2-Bac was shown as reliable for detecting dog, horse, sheep, bird, gull, ruminant, and pig fecal contamination sources, respectively, in Finnish watersheds. Still, all mtDNA targets and the Pig-2-Bac marker were not detected in surface water samples.
• The sensitivity of the human-specific marker HF183 was 100% with RNA-based and DNA-based approaches. However, the specificity and accuracy of the marker were higher with the DNA-based approach (95–96%) than with the RNA-based approach (54–56%). Despite the cross-reactivity, the GC values were many folds higher in targeted sewage samples than in non-targeted animal fecal samples. Therefore, the use of RNA as a template for the HF183 assay in the future could be justified when employed together with a DNA template.
• The general fecal marker GenBac3 had a higher detection rate and GC in studied mammal fecal materials than in bird fecal materials. It may indicate that measuring the marker targeted to general *Bacteroidales* may not cover the fecal contamination from bird species.

## Data Availability Statement

The raw data supporting the conclusions of this article will be made available by the authors, without undue reservation.

## Author Contributions

AR, A-MH, TT, and TP contributed to conceptualization and design of the study. A-MH, SU, and TT organized the sampling. AR executed the laboratory study and the original data calculations and wrote the first draft of the manuscript. AT performed the statistical analysis under the supervision of AV and wrote major parts of the results, the first draft of the discussion, and visualized the data. TP supervised the work and was in charge of the funding acquisition. All authors contributed to manuscript editing, read, and approved the final version.

## Conflict of Interest

The authors declare that the research was conducted in the absence of any commercial or financial relationships that could be construed as a potential conflict of interest.
