# Supplementary material for: The Use of Ribosomal RNA as a Microbial Source Tracking Target Highlights the Assay Host-Specificity Requirement in Water Quality Assessments
Source: Front Microbiol. 2021 Jun 2;12:673306. doi: 10.3389/fmicb.2021.673306 (PMC8206488; doi:10.3389/fmicb.2021.673306)
Supplement: Supplementary file 1 [file Data_Sheet_1.docx]

The use of ribosomal RNA as a microbial source tracking target highlights the assay host-specificity requirement in water quality assessments

Supplementary Material

# Supplementary material 1: Description of sampling locations

Most of the fecal and water samples were collected from rural sites and bathing areas in a municipality in Norther Ostrobothnia, Finland (1-4). Sampling site 1 was an EU bathing area on Bothnia Bay coast with usually good quality waters and a boat dock nearby. No pastures were located nearby. This site was handled as a control site among the sampling sites in this area.

Sampling site 2 with three subsites (2A-C) was a small public bathing area on Bothnia Bay coast with nearby boat dock and pasture areas for sheep and cattle. Both sheep and cattle had access to sea water, but sheep had drinking water points elsewhere at their pasture, whereas cows drank only brackish coastal water of Baltic Sea with low salinity. A small river collecting runoffs from pastures and fields flows to the bay at this area. A shoal nested by waterfowls was situated near the bathing area. The samples were taken on the bathing area (2A), from the sea near the cattle pasture where water level changes may occasionally raise water on the pasture (2B) and at the opposite side of a ness from the bathing area (2C). The water rising at the site 2B was not observed during the summer 2018. Sixteen cattle fecal samples, 26 bird fecal samples and 19 sheep fecal samples were collected from this area for method development.

The sampling site 3 with three subsites (3A-C) was a public bathing area on Bothnia Bay coast with nearby pasture areas for cattle. A river flowing through rural area into the Bothnia Bay situated at the sampling area. The samples were taken on the bathing area (3A), from the river before the estuary (3B) and upriver near settlement (3C).

Sampling site 4 with three subsites (4A-C) was a small bay on Bothnia Bay coast with a private holiday cottage near pastures and a migratory bird nesting area. A boat dock was located at the opposite side of the bay. At pasture, cattle had direct access to water. The samples were taken from the beach at the private cottage (4A), from the shore near the pastured area (4B) and from a ditch flowing into the bay (4C).

Sampling sites 5 and 6 were located in a city in Northern Ostrobothnia, Finland. Sampling site 5 was a sewage treatment plant handling the municipal sewage from the city. The samples were taken from the effluent water. Sampling site 6 was a trench surrounded by green space and for example a dog park and a horse stable in suburb area. Fecal samples from a dog, a horse and a mixed sample from several bird feces were collected from the area for method development.

Sampling sites 7 and 8 were located in a city in Northern Savonia, Finland. Sampling site 7 was a small creek in a park at the city center. Sampling site 8 was a culvert passing under a walking path surrounded by horse pastures in suburb area. Two horse fecal samples were collected from the site 8 for method development. To complete the sample collection for the method development, horse fecal samples were collected from a riding school located in the municipality of Siilinjärvi (Northern Savonia region).

Sampling site 9 was an EU bathing area in a small city in Pirkanmaa, Finland. The site was located near city center and surrounded by green space. During summer 2018, a lot of barnacle gooses were settled in the park and the bathing area. Thirteen goose fecal samples were collected for method development. An accidental sewer leakage had occurred in the area approximately ten days before the sampling.

Sampling sites 10,11, 12 and 13 were located in a city in Pirkanmaa, Finland. Sampling site 10 was a trench collecting runoff waters from large suburban area. Sampling site 11 was a trench in which the trench 10 flows, and which is running through vegetable plots. Sampling site 12 was a trench collecting runoff water large municipal and industrial areas. A significant walking path highly used by dog walkers leads near the site 12. High levels of fecal bacteria have been identified from all three sampling sites during previous years. Sampling site 13 was a spring in a park at suburb area. A lot of ducks live in the area and fecal bacteria has been identified from this site as well. A duck fecal sample was collected from the site 13 for method development.

Multiple sites were sampled in Kanta-Häme area, Finland (14-20). Sampling site 14 with five subsites (A-E) was a trench running through a municipal area and fields from site A to E. The sampling site 14A collects runoff water from large municipal area. The trench resembles wetland at this site and a lot of ducks live there. Five dog, two waterfowl, one other bird and one hare fecal samples were collected from this site for method development. At the sampling site 14B the trench runs through a stretch of field, which is used by dog walkers. Runoff water from a horse farm and probably from the municipal area may end up in this site. Four dog and one bird fecal samples were collected from the site 14B for method development. The trench runs through a municipal area between the sampling sites 14C and 14D. Misconnected sewers had been found at the site 14D, which may have affected the quality of runoff waters. Five dog, and one hare fecal sample were collected from the site 14C, and one bird and five dog fecal samples from the site 14D for method development. Before the sampling site 14E, the trench runs through a field area. At the sample site there was a thicket, where deer may thrive. Three bird fecal samples were collected from this site for method development.

Sampling site 15 was a horticultural school growing woody plants, such as apple trees, where surface water from a shallow stream was used for irrigation. The subsites for the site 15 were 15A, where the samples were taken before exposure to LED-ultraviolet light (LED-UV device by LedFuture Inc., Kuopio, Finland), and 15B, where the water was disinfected with LED-UV before sampling.

Sampling site 16 was a sewage treatment plant using activated sludge as secondary treatment. The subsites for the site 16 were 16A, where the sample was taken from the effluent; 16B, where the sample was taken from the effluent treated with LED-UV; and 16C was effluent after a wetland treatment. Sampling site 17 was surface water from an effluent discharge area of another sewage treatment plant at the same geographical area.

Sampling sites 18-20 were surface water sites situated near animal farms. The site 18 was trench near a swine farm and a large area of fields. The site 19 was a runoff water tank with biocarbon filters in a horse stable, where the runoff from the paddocks was collected. The site 20 was a trench surrounding paddocs at a large horse farm.

Table S1. Wastewater and surface water samples included in the study. Sites 1-6: Northern Ostrobothnia; sites 7-8: Northern Savonia; sites 9-13: Pirkanmaa; sites 14-20: Kanta-Häme.

| Sample type | Sampling site | Water type | No of samples | Total |
| --- | --- | --- | --- | --- |
| Surface water, bathing area | 1 | Brackish | 5 | 21 |
|  | 2A | Brackish | 5 |  |
|  | 3A | Brackish | 5 |  |
|  | 4A | Brackish | 5 |  |
|  | 9 | Fresh | 1 |  |
| Surface water, rural | 2B | Brackish | 5 | 34 |
|  | 2C | Brackish | 5 |  |
|  | 3B | Fresh | 5 |  |
|  | 3C | Fresh | 5 |  |
|  | 4A | Brackish | 5 |  |
|  | 4B | Fresh | 5 |  |
|  | 18 | Fresh | 1 |  |
|  | 19 | Fresh | 1 |  |
|  | 20 | Fresh | 2 |  |
| Surface water, urban | 6 | Fresh | 5 | 25 |
|  | 7 | Fresh | 3 |  |
|  | 8 | Fresh | 3 |  |
|  | 10 | Fresh | 1 |  |
|  | 11 | Fresh | 1 |  |
|  | 12 | Fresh | 1 |  |
|  | 13 | Fresh | 1 |  |
|  | 14A | Fresh | 2 |  |
|  | 14B | Fresh | 2 |  |
|  | 14C | Fresh | 2 |  |
|  | 14D | Fresh | 2 |  |
|  | 14E | Fresh | 2 |  |
| Garden irrigation system | 15A | Fresh | 3 | 5 |
|  | 15B | Fresh, LED-UV-treated | 2 |  |
| Sewage effluent | 5 | Sewage effluent | 1 | 10 |
|  | 16A | Sewage effluent | 4 |  |
|  | 16B | Sewage effluent, LED-UV treated | 2 |  |
|  | 16C | Sewage effluent, wetland treated | 2 |  |
|  | 17 | Sewage effluent | 1 |  |

Table S2. The qPCR conditions used in sample analysis in assays with TaqMan and SYBR Green chemistry.

|  | TaqMan Environmental Assays | | SYBR Green Environmental Assays | |
| --- | --- | --- | --- | --- |
| Enzyme Activation | 10 min | 95°C | 10 min | 95°C |
|  | 40x | | 40x | |
| Denaturation | 15 s | 95°C | 15 s | 95°C |
| Annealing | 1 min | 60°C | 1 min | 60°C |

Table S3. Performance features of the qPCR runs conducted in the study.

| Assay | Range of RNA blanks^1^ | Range of DNA blanks^1^ | Limit of detection^2^ | Range of amplification efficiency (%) | Range of R^2^ | Range of quantification (copies) |
| --- | --- | --- | --- | --- | --- | --- |
| GenBac3 | 0-60 | 0-3 | 3 | 80.4-111.3 | 0.954-0.999 | 20-200 000 |
| HF183 | 0 | 0 | 3 | 82.5-103.3 | 0.969-0.999 | 20-200 000 |
| BacCan | 0 | 0 | 3 | 88.3-100.2 | 0.976-0.998 | 200-200 000 |
| Rum-2-Bac | 0-8 | 0 | 3 | 81.3-108.8 | 0.971-0.999 | 20-200 000 |
| Pig-2-Bac | 0 | 0 | 3 | 81.7-106.1 | 0.983-0.999 | 20-200 000 |
| Gull4 | 0 | 0 | 3 | 83.0-105.6 | 0.957-0.998 | 20-200 000 |
| GFD | 0-71 | 0 | 3 | 84.3-106.3 | 0.978-0.999 | 20-200 000 |
| Av4143 | 0 | 0 | 3 | 72.9-110.5 | 0.946-0.999 | 20-200 000 |
| DogND5 | - | 0-9 | 3 | 84.4-107.2 | 0.988-0.998 | 20-200 000 |
| SheepCytB | - | 0 | 3 | 82.2-109.0 | 0.964-1.000 | 20-200 000 |
| HorseCytB | - | 0 | 3 | 81.3-102.6 | 0.970-0.999 | 20-200 000 |
| ^1^Range of copy numbers per reaction (copies/rxn) in negative control samples. ^2^Limit of detection set according to Bustin et al. 2009. | | | | | | |

# Supplementary Material 2. Data calculation equations of qPCR analysis

Nucleic acid (NA) factor corresponds to the complementary DNA (cDNA) and the DNA factor, depending on the raw data handled.

**Fecal samples:**

$$LOD \left( \frac{GC}{100 mg} \right)=0.1*\frac{Detection limit\left( \frac{GC}{rxn} \right)}{Sample mass \left( g \right)*NA factor}$$

$$LOQ \left( \frac{GC}{100 mg} \right)=0.1*\frac{Detection limit \left( \frac{GC}{rxn} \right)+Quantification limit \left( \frac{GC}{rxn} \right)}{Sample mass \left( g \right)*NA factor}$$

$$NA \left( \frac{GC}{100 mg} \right)=0.1*\frac{Clean NA}{Sample mass \left( g \right)*NA factor}, \mathrm{where}$$

$$Clean NA \left( \frac{GC}{rxn} \right)=NA result \left( \frac{GC}{rxn} \right)-detection limit \left( \frac{GC}{rxn} \right)$$

$$cDNA factor=Dilution in TURBO cleaning*Subsample to RT*Subsample to qPCR$$

$$DNA factor=Subsample to qPCR$$

**Water samples:**

$$LOD \left( \frac{GC}{100 ml} \right)=100*\frac{Detection limit\left( \frac{GC}{rxn} \right)}{Sample volume \left( ml \right)*NA factor}$$

$$LOQ \left( \frac{GC}{100 ml} \right)=100*\frac{Detection limit \left( \frac{GC}{rxn} \right)+Quantification limit \left( \frac{GC}{rxn} \right)}{Sample volume \left( ml \right)*NA factor}$$

$$NA \left( \frac{GC}{100 ml} \right)=100*\frac{Clean NA}{Sample volume \left( ml \right)*NA factor}, \mathrm{where}$$

$$Clean NA \left( \frac{GC}{rxn} \right)=NA result \left( \frac{GC}{rxn} \right)-detection limit \left( \frac{GC}{rxn} \right)$$

$$cDNA factor=Subsample to TURBO cleaning*Dilution in TURBO cleaning*Subsample to RT*Subsample to qPCR$$

$$DNA factor=Subsample to qPCR$$

Table S4. The detection rate and GC count of assays on targeted fecal materials or sewage samples with RNA-based and DNA-based approaches. Mann-Whitney test compared the GC quantification and McNemar test compared detected frequency between DNA-based and RNA-based approach. NA = Not applicable.

| Assay | RNA detection rate (%) | **RNA Mean ± SE, Median (GC / 100 mg or ml)** | DNA detection rate (%) | **DNA Mean ± SE, Median (GC / 100 mg or ml)** | Statistical significance and test |
| --- | --- | --- | --- | --- | --- |
| GenBac3  (n = 151) | 79.5 | 9.67±0.17; 10.35 | 74.8 | 7.85±0.17; 8.19 | *p* < 0.001, Mann-Whitney U Test; *p* = 0.206, McNemar test |
| HF183  (n = 8) | 100 | 8.00±0.17; 8.00 | 100 | 5.73±0.13; 5.75 | *p* < 0.001, Mann-Whitney U Test |
| BacCan  (n = 21) | 90.5 | 10.62±0.52; 11.34 | 76.2 | 7.10±0.23; 6.84 | *p* < 0.001, Mann-Whitney U Test; *p* = 0.205, McNemar test |
| Rum-2-Bac  (n = 35) | 100 | 9.71±0.15; 9.67 | 100 | 8.53±0.11; 8.55 | *p* < 0.001, Mann-Whitney U Test |
| Pig-2-Bac  (n = 6) | 100 | 9.77±0.16; 9.84 | 100 | 8.12±0.17; 7.97 | *p*  = 0.002, Mann-Whitney U Test |
| Gull4  (n = 17) | 100 | 8.98±0.23; 9.57 | 100 | 6.87±0.21; 7.32 | *p* < 0.001, Mann-Whitney U Test |
| GFD  (n = 73) | 88.1 | 7.50±0.15; 7.49 | 65.7 | 4.70±0.17; 4.48 | *p* < 0.001, Mann-Whitney U Test; p = 0.002, McNemar test |
| Av4143  (n = 67) | 65.7 | 6.71±0.21; 6.60 | 62.7 | 5.15±0.18; 5.21 | *p* < 0.001, Mann-Whitney U Test; *p* = 0.429, McNemar test |
| DogND5  (n = 21) | NA | NA | 95 | 6.14±0.16; 6.07 | NA |
| HorseCytB  (n = 19) | NA | NA | 100 | 5.67±0.17; 5.89 | NA |
| SheepCytB  (n = 19) | NA | NA | 100 | 8.27±0.08; 8.27 | NA |

.

**Figure S1. GenBac3 GC from fecal samples from different hosts.** The p-value is based on the Mann-Whitney U test when the median value of pooled birds and mammal fecal samples (> LOQ) are compared. uBird = unidentified species of birds. Duck and waterfowl samples were pooled together for the boxplot as the number of > LOQ samples from these two groups of hosts was low.

**Table S5. Gene copy numbers of the RNA-based and DNA-based MST marker assays in fecal samples in multiple targeted host groups.** If a marker is targeted for multiple hosts: Host I, Host II and Host III). uBird = Unknown birds

| **Markers** | **Mean ± SE, Median (GC / 100 mg)** | | | **p-value and statistical test** |
| --- | --- | --- | --- | --- |
|  | **Host I** | **Host II** | **Host III** |  |
| **Rum-2-Bac**  **RNA** | Cow (n= 16), 10.28 ±0.18, 10.60 | Sheep (n=19), 9.22±0.15, 9.39 | - | ***P* < 0.001**, Mann-Whitney U test (Cow > Sheep) |
| **Rum-2-Bac**  **DNA** | Cow (n= 16), 8.11 ±0.13, 8.16 | Sheep (n=19), 8.90±0.12, 8.87 | - | ***P* < 0.001**, Mann-Whitney U test (Sheep > Cow) |
| **Gull4 RNA** | Gull (n=17), 9.87±0.21, 9.85 | uBird (n= 27), 8.42±0.30, 8.64 | - | ***P* < 0.001**, Mann-Whitney U test (Gull > uBirds) |
| **Gull4 DNA** | Gull (n=17), 7.21±0.27, 7.37 | uBird (n= 26), 6.74±0.29, 7.22 | - | *P=* 0.518*,* Mann-Whitney U test |
| **GFD**  **RNA** | uBird (n= 29), 7.64 ±0.21, 7.68 | Gull (n=15), 7.31±0.38, 7.48 | Goose (n=13), 7.34±0.30, 7.37; Duck & waterfowl (n=3), 7.70 ±0.43, 7.45 | *P =* 0.873, Kruskal Wallis test |
| **GFD**  **DNA** | uBird (n= 24), 5.04 ±0.23, 4.89 | Gull (n=7), 4.06±0.30, 3.55 | Goose, duck & waterfowl (n=6), 4.18±0.20, 4.07 | *P =*  0.073, Kruskal Wallis test |
| **Av4143**  **RNA** | uBird (n= 25), 6.38 ±0.31, 6.00 | Gull (n=16), 7.30±0.31, 7.51 | Goose, duck & waterfowl (n=4), 6.04±0.16, 6.10 | *P =* 0.253, Kruskal Wallis test |
| **Av4143**  **DNA** | uBird (n= 23), 5.03 ±0.23, 5.17 | Gull (n=14), 5.56±0.30, 5.42 | Goose, duck & waterfowl (n=3), 4.18±0.53, 3.70 | *P =* 0.786, Kruskal Wallis test |

Table S6. The detection rate of the species-specific assays in surface water samples with RNA-based and DNA-based approaches.

| Assay | RNA detection rate (%) | DNA detection rate (%) | Statistical significance and test |
| --- | --- | --- | --- |
| GenBac3 (n = 95) | 99 | 100 | *p* = 1.00 , McNemar test |
| HF183 (n = 95) | 52.1 | 39.6 | *p* = 0.082 , McNemar test |
| BacCan | Not done | Not done | *Not applicable* |
| Rum-2-Bac (n = 95) | 44.8 | 10.5 | *p* < 0.001, McNemar test |
| Pig-2-Bac (n = 95) | Not detected | Not detected | *Not applicable* |
| Gull4 (n = 95) | 71.8 | 43.6 | *p* < 0.001, McNemar test |
| GFD (n = 95) | 88.5 | 10.5 | *p* < 0.001, McNemar test |
| Av4143 (n = 95) | 49.0 | 10.5 | *p* < 0.001, McNemar test |

**Figure S2. GenBac3 GC in the groups of surface water samples and analysed using RNA-based and DNA-based approach.** In x-axis sample groups. w. = water, f. = fresh, b.= brackish, n = number of data > limit of quantification and used for boxplot analysis.

Figure S3. HF183 GC in the groups of surface water samples and analysed using RNA-based and DNA-based approach. In x-axis sample groups. w. = water, f. = fresh, b.= brackish, n = number of data > limit of quantification and used for boxplot analysis.

**Figure S4. Rum-2-Bac GC in the groups of surface water samples and analysed using RNA-based and DNA-based approach.** In x-axis sample groups. w. = water, f. = fresh, b.= brackish, n = number of data > limit of quantification and used for boxplot analysis.

**Figure S5. Gull4 GC in the groups of surface water samples and analysed using RNA-based and DNA-based approach.** In x-axis sample groups. w. = water, f. = fresh, b.= brackish, n = number of data > limit of quantification and used for boxplot analysis.

**Figure S6. GFD GC in the groups of surface water samples and analysed using RNA-based and DNA-based approach.** In x-axis sample groups. w. = water, f. = fresh, b.= brackish, n = number of data > limit of quantification and used for boxplot analysis.

**Figure S7. Av4143 GC in the groups of surface water samples and analysed using RNA-based and DNA-based approach.** In x-axis sample groups. w. = water, f. = fresh, b.= brackish, n = number of data > limit of quantification and used for boxplot analysis.

**Figure S8. DogND5 GC in the groups of surface water samples and analysed using mtDNA-based approach.** In x-axis sample groups. w. = water, f. = fresh, b.= brackish, n = number of data > limit of quantification and used for boxplot analysis.
